# A Personalized Risk Model for Azacitidine Outcome in Myelodysplastic Syndrome and Other Myeloid Neoplasms Identified by Machine Learning Model Utilizing Real-World Data

**DOI:** 10.3390/cancers15164019

**Published:** 2023-08-08

**Authors:** Kirsty Sharplin, William Proudman, Rakchha Chhetri, Elizabeth Ngoc Hoa Tran, Jamie Choong, Monika Kutyna, Philip Selby, Aidan Sapio, Oisin Friel, Shreyas Khanna, Deepak Singhal, Michelle Damin, David Ross, David Yeung, Daniel Thomas, Chung H. Kok, Devendra Hiwase

**Affiliations:** 1 Royal Adelaide Hospital, Central Adelaide Local Health Network, Adelaide, SA 5000, Australia; 2 Precision Medicine Theme, South Australian Health and Medical Research Institute (SAHMRI), Adelaide, SA 5000, Australia; 3 Adelaide Medical School, The University of Adelaide, Adelaide, SA 5000, Australia; 4 Beaumont Hospital, D09 V2N0 Dublin, Ireland; 5 Centre for Cancer Biology, University of South Australia and SA Pathology, Adelaide, SA 5000, Australia; 6 Genetic and Molecular Pathology, SA Pathology, Adelaide, SA 5000, Australia

**Keywords:** azacitidine, prognostication, MDS, survival, machine learning

## Abstract

**Simple Summary:**

Myelodysplastic syndrome (MDS) is one of the most-common blood cancers in older individuals. Although azacitidine is the most-commonly used treatment for MDS, only 30–40% of patients respond to it, and responses may not be achieved up to six cycles of treatment. Moreover, there are no universally accepted prognostic models that will identify patients who are unlikely to benefit. To address this shortcoming, we used a machine learning model (“Artificial Intelligence”) to identify patients who are unlikely to benefit from azacitidine. Our study provides a machine learning model that predicts patients who are less likely to benefit from azacitidine. The median survival of Poor-risk patients was only 8 months compared to 23 months in the favorable risk group. Importantly the model can be used during routine practice not only in major hospitals, but also in small community practice.

**Abstract:**

Azacitidine is an approved therapy for higher-risk myelodysplastic syndrome (MDS). However, only 30–40% patients respond to azacitidine, and the responses may take up to six cycles to become evident. Delayed responses and the myelosuppressive effects of azacitidine make it challenging to predict which patients will benefit. This is further compounded by a lack of uniform prognostic tools to identify patients at risk of early treatment failure. Hence, we performed a retrospective analysis of 273 consecutive azacytidine-treated patients. The median overall survival was 16.25 months with only 9% alive at 5 years. By using pre-treatment variables incorporated into a random forest machine learning model, we successfully identified those patients unlikely to benefit from azacytidine upfront (7.99 vs. 22.8 months, *p* < 0.0001). This model also identified those who required significantly more hospitalizations and transfusion support. Notably, it accurately predicted survival outcomes, outperforming the existing prognostic scoring system. By integrating somatic mutations, we further refined the model and identified three distinct risk groups with significant differences in survival (5.6 vs. 10.5 vs. 43.5 months, *p* < 0.0001). These real-world findings emphasize the urgent need for personalized prediction tools tailored to hypomethylating agents, reducing unnecessary complications and resource utilization in MDS treatment.

## 1. Introduction

Myelodysplastic syndromes (MDSs) are characterized by ineffective hematopoiesis and a risk of progression to acute myeloid leukemia (AML) [1]. Without treatment, overall survival (OS) for higher-risk MDS is poor [2,3]. The hypomethylating agent (HMA) azacitidine improves cytopenia, prolongs survival, and forestalls AML transformation [4,5].

The pivotal AZA-001 trial achieved a median OS of 24.5 months in azacitidine-treated patients compared to 15 months in patients managed with conventional care (*p* = 0.0001). The two-year probability of survival was 50.8% for azacitidine compared to 26.2% in the conventional care group [6]. However, several real-world registry studies suggest that the median OS benefit with azacitidine is much lower, ranging between 11 and 16 months [1,7,8,9,10,11,12,13]. Furthermore a survival benefit of only three months was observed in patients with MDS-excess blasts, a proxy for higher-risk MDS [14]. Current recommendations state that six cycles are required prior to declaring treatment failure. This prolonged time to response, coupled with the myelosuppressive effects, make the timely evaluation of treatment to predict which patients will ultimately benefit difficult. When coupled with low response rates (30–40%), patients may be receiving a treatment that is ultimately futile with the additional risk of treatment-related complications [4]. This is further compounded by the lack of upfront prognostic tools to identify patients most likely to benefit from hypomethylating agents versus those that are at high risk of treatment failure. We hypothesized that a machine learning approach examining baseline clinical and molecular biomarkers will identify patients unlikely to benefit and at higher risk of failure. We also characterized healthcare utilization and transfusion burden in azacitidine-treated patients.

## 2. Materials and Methods

### 2.1. Patients

A total of 273 consecutive patients enrolled in the South Australian MDS (SA-MDS) registry diagnosed with either MDS, chronic myelomonocytic leukemia (CMML), or oligoblastic AML and treated with azacitidine as the frontline therapy between June 2004 and February 2020 were included. The SA-MDS registry is a multi-center collaboration between 3 major treatment centers within South Australia. The diagnosis was based on World Health Organization 2016 criteria and included therapy-related MDS and therapy-related CMML. This study was conducted in accordance with the Declaration of Helsinki Principles with patients providing informed consent or exemption approval from the local ethics committee.

All patients included received azacitidine monotherapy, administered at a dose of 75 mg/m^2^ for 7 days on a 5 + 2 + 2- or 7-day schedule. Information about the total number cycles and delay between the cycles was collected. A delay in the cycle was defined as an interval greater than 35 days between cycles.

### 2.2. Response Assessment

Response assessments were carried out at the time of bone marrow biopsy and at 6, 12, 18, 24, and 36 months after starting azacitidine. Where a bone marrow biopsy was not performed, only hematological responses were assessed. Responses were defined as per the International Working Group (IWG) response criteria for myelodysplasia [15] and included complete remission (CR), partial remission (PR), bone marrow CR (bmCR), hematological improvement (HI), and stable disease (SD). HI was only assessed in those patients who required transfusion and had cytopenia at diagnosis. We also grouped responses at six months into three groups: (i) responders, which included CR, PR, HI, and bmCR with or without HI; (ii) stable disease; (iii) non-responders, which included patients who failed therapy or progressed.

### 2.3. Construction of Prediction Model Using Random Survival Forest Machine Learning Algorithm

To identify predictors for patients who are less likely to benefit from azacitidine, we employed a random survival forest algorithm (RSF) [16] as the data type was survival analysis with censoring. Briefly, 273 patients were randomly allocated to the training and validation cohorts with a 1:1 ratio based on an equal proportion of IPSS-R categories. The training cohort (*n* = 139) was subjected to RSF using 40 clinical variables that were measured before starting azacitidine. Top-ranking variables were identified by RSF based on variable importance analysis using the training cohort. The cross-validated prediction error was measured by Harrell’s concordance index [17] to assess prediction performance. The cross-validated mortality risk score [16] derived from the RSF model in the training cohort was used to divide patient samples into Poor risk and Standard risk based on maximally selected rank statistics. In addition, the performance of the constructed RSF model was further assessed using the independent validation cohort (*n* = 134) to ensure robustness and generalization of the constructed predictive model. The assessment of prediction performance for the validation cohort was based on Harrell’s concordance index. For more details and the process of RSF, please refer to the Supplementary Methods and Supplementary Figure S1.

### 2.4. Statistical Analysis

Descriptive statistics were used to describe patient and disease characteristics, and disease was categorized based on the International Prognostic Scoring System score [12]. Intergroup comparisons were carried out using Fisher’s exact test for categorical variables and the Wilcoxon rank sum test or Student’s t-test for continuous variables. Overall survival (OS) was calculated from commencement of azacitidine to death from any cause or end of follow-up. To optimize the reliability of long-term survival assessment and ensure five-year follow-up from commencement of azacitidine, we analyzed the survival of patients who commenced azacitidine before May 2016 and were followed until death or end of follow-up on 30 May 2021. Survival probabilities over time were estimated using the Kaplan–Meier methodology, and comparisons of survival across subgroups were conducted using the two-sided log-rank test. The optimal cut-point for selected continuous variables was determined using the maximally selected rank statistics. Multivariable models of overall survival were performed with Cox proportional hazards regression. Hazard ratios and 95% confidence intervals (CIs) are reported for covariates, along with *p*-values from the Wald test. Multivariate gene mutation selection based on Cox proportional regression analysis with Lasso regularization and three-fold cross-validation was performed for genes mutated in >5% of cases. *p*-values < 0.05 were considered statistically significant. All statistical analyses were conducted using the R statistical platform (https://www.r-project.org/, accessed on 30 March 2023) v.4.1.1 and GraphPad Prism v.9.2.0.

## 3. Results

At the time of analysis, 273 consecutive patients were treated with azacitidine. Their median age at diagnosis was 73 (interquartile range 67–78) years, and 40% (*n* = 111) of patients were ≥75 years of age. The majority were male (70%), and 78% were de novo MDS or oligoblastic AML, while 22% were therapy-related (*n* = 59). Of the evaluable 185 de novo MDS/oligoblastic AML patients treated with azacitidine, 97% (*n* = 180) were classified as Intermediate, High, and Very High risk and only 3% (*n* = 5) were classified as Low or Very Low risk at diagnosis (Tables S1 and S2).

The median time from diagnosis to starting azacitidine was significantly shorter in patients with IPSS-Intermediate-2 and High risk compared to those in the Low and Intermediate-1 group (1.3 months vs. 23.1 months; *p* < 0.001), which reflects clinical practice in Australia where azacitidine is approved only for higher-risk patients. For the lower-risk patients who progressed to higher risk, the median interval between becoming eligible and starting azacitidine was only 1.1 months (95% CI: 1.01–1.32).

**Figure 1:**
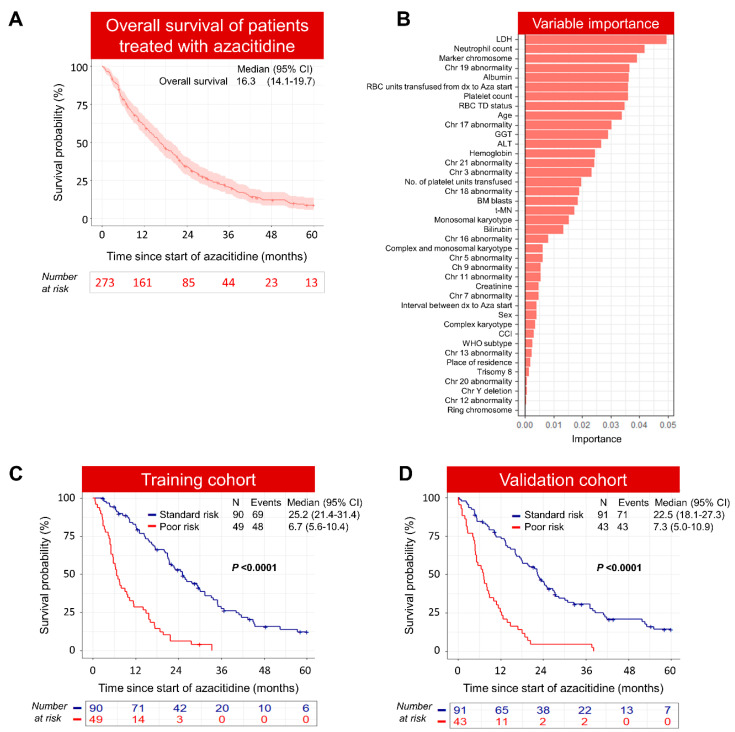
Machine learning model identifies Poor risk patients who are unlikely to benefit from azacitidine therapy. (**A**) Median OS of azacitidine-treated patients. Shaded area represents 95% confidence interval. (**B**) Variable importance analysis showing the top variables from the most to the least importance that affect overall survival of azacitidine-treated patients. (**C**) Significant overall survival difference between Poor- and Standard-risk patients in the training cohort. (**D**) Validation cohort confirming the significant difference in OS in Poor- and Standard-risk patients.

At the time of analysis, median follow-up for the entire cohort was 24.1 months (range 12.4–41.9) with only 10 patients still on treatment; 12% (*n* = 32) were alive, and censoring for allogeneic stem cell transplant, the median OS was 16.3 (95% CI 14.1,19.7) months (Figure 1A). The estimated three-year and five-year OS of patients based on long-term follow-up data (treated prior to August 2016) was only 20% (95% CI 16–26%) and 9% (95% CI 6–13%), respectively.

### 3.1. Personalized Risk Model to Predict Outcome of Patients Treated with Azacitidine

To identify predictors for patients who are less likely to benefit from azacitidine, we first randomly allocated 273 patients into training and validation cohorts with a 1:1 ratio based on an equal proportion of the IPSS-R. Patient characteristics for the training cohort and independent validation cohort are shown in Table S3.

We used a random forest algorithm to predict the survival of MDS patients treated with azacitidine using a training cohort (*n* = 139), based on twenty routine clinical and twenty cytogenetic variables measured before starting azacitidine (Table S4). The schematic of the model development process is shown in Supplementary Figure S1A. The variable importance analysis results based on the training cohort indicated that LDH, neutrophil count, albumin, abnormal chromosome 19, marker chromosome, platelet count, and RBC-TD status were the most-influential variables predicting the survival of azacitidine-treated patients (Figure 1B). The model classified forty-nine patients (35%) into the Poor-risk group and ninety patients (65%) into the Standard-risk group. As expected, the median OS for the Poor-risk group was significantly inferior compared to the Standard-risk group (6.7 vs. 25.2 months; *p* < 0.0001) (Figure 1C). Harrel’s c-index in this training cohort was 0.72 (Supplementary Figure S1B). Importantly, the model was further validated in an independent cohort (*n* = 134) with a Harrell’s c-index of 0.72, similar to the training cohort (Supplementary Figure S1B). In the validation cohort, the model classified forty-three (32%) patients into the Poor-risk group and ninety-one (68%) patients into the Standard-risk group. The median OS was significantly inferior in the Poor-risk group compared to the Standard-risk group (7.3 vs. 22.5 months; *p* < 0.0001) (Figure 1D). Taken together, Poor-risk patients predicted by our model had a shorter median survival time compared to Standard-risk patients treated with azacitidine.

To determine variables with predictive capacity for OS on azacitidine, the top five variables selected by the model were included in univariable models for both the validation and training cohorts.

**Figure 2:**
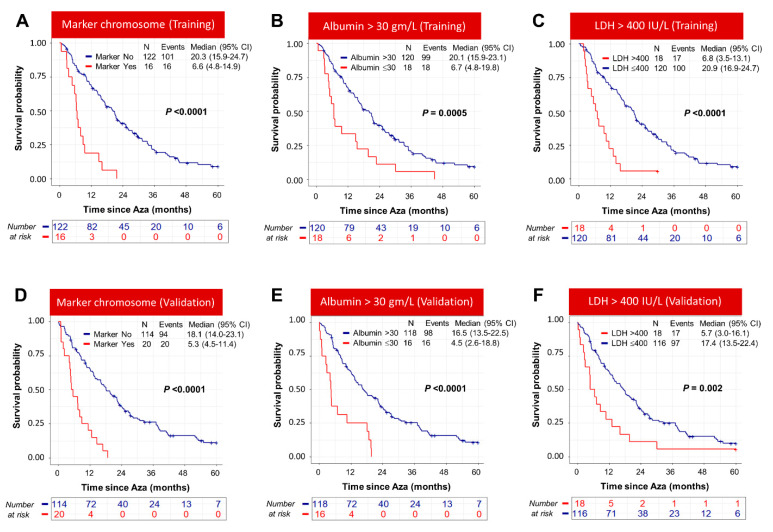
Baseline clinical parameters identified by the machine learning model influence overall survival of MDS patients treated with azacitidine. In both the training and validation cohort, marker chromosome (**A**,**D**), albumin (**B**,**E**), and LDH (**C**,**F**) predicted overall survival of azacitidine-treated patients.

In the training cohort, the overall survival of patients with albumin <31 gm/L, LDH > 400 IU/L, neutrophil counts, marker chromosome, and chromosome 19 abnormality was significantly poor compared to patients without these abnormalities (Figure 2A–C and Supplementary Figure S2A,B). All variables except for neutrophil count and chromosome 19 abnormality were associated with significantly poor OS in the validation cohort, which could be due to the small sample size for chromosome 19 abnormality (*n* = 7) (Figure 2D–F and Supplementary Figure S2C,D). In order to seek further insight, we compared the mean neutrophils counts and chromosome 19 abnormalities in different risk groups of MDS, AML, t-MN, and CMML. There was no significant difference in the average neutrophil count prior to starting azacitidine and abnormal chromosome 19 between the groups (Supplementary Results).

**Figure 3:**
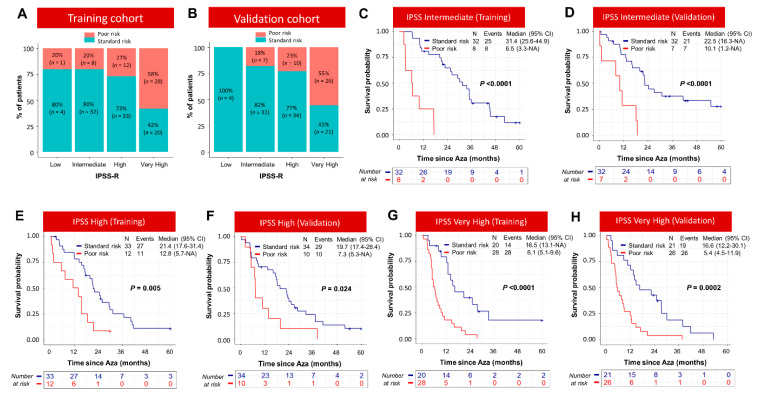
Poor-risk group, identified by the machine learning model, stratified IPSS-R groups. (**A**,**B**) Standard- and Poor-risk groups, identified by the machine learning model, are distributed across the IPSS-R risk groups. In the training and validation cohorts, Poor-risk group stratified IPSS-R Intermediate- (**C**,**D**), High- (**E**,**F**), and Very-High- (**G**,**H**) risk groups further with significant survival difference.

We next compared the performance of the model with that of IPSS-R for predicting overall survival of azacitidine-treated patients. Our model concordance index (c-Index), a commonly used metric to evaluate how good a prediction model is, was 0.72 in the training and validation cohort, while it was only 0.62 and 0.61 for the widely used IPSS-R model in the training and validation cohorts (Supplementary Figure S3A). The OS for IPSS-R in both the training and validation cohorts is shown in Supplementary Figure S3B,C. We observed that Poor-risk patients, identified by the model, were distributed across the IPSS-R risk group in both the training and validation cohorts (Figure 3A,B), and importantly, it stratified IPSS-R Intermediate, High, and Very High risk groups further with significant survival differences in both the training and validation cohorts (Figure 3C–H).

### 3.2. Integration of Somatic Mutation Information into the Prognostic Model Based on Clinical Variables Identified Three Distinct Groups of Patients with Significant Survival Differences

**Figure 4:**
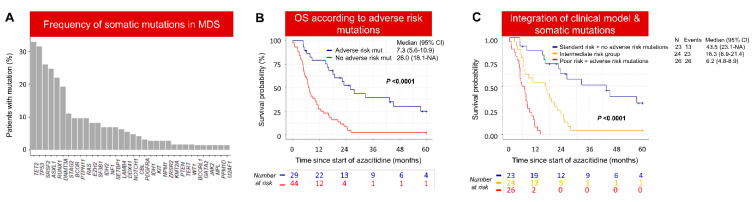
Integration of clinical variables and somatic mutation profile can identify patients who are less likely to benefit from azacitidine therapy. (**A**) Frequency of somatic mutations in MDS patients treated with azacitidine (*n* = 73). (**B**) OS was significantly poor in patients with Adverse risk mutations compared to patients without these mutations. (**C**) Integration of clinical variables and somatic mutations defines three distinct risk groups with significant survival difference. Intermediate-risk group includes Poor-risk without Adverse risk mutations and Standard-risk group with Adverse risk mutations. The other two groups are Poor-risk group with Adverse risk mutations and Standard-risk group without Adverse risk mutations.

Mutation data were available for 73 patients, with the relative frequency outlined in Figure 4A. A high incidence of *TP53* mutations (*TP53*^mut^) is reflective of the inclusion of t-MDS patients. The median OS of patients with *TP53* wild-type (*TP53*^wt^) was significantly longer compared to *TP53*^mut^ (17.40 vs. 6.91; *p* < 0.0001) (Supplementary Figure S4A). To identify mutations associated with poor outcome in *TP53*^wt^ cases, feature selection was performed in genes mutated in >5% of cases as described in the Statistical Analysis Section. As a result, mutations in *BCOR/BCORL1, SETBP1, RAS, EZH2*, or *DNMT3A* were associated with inferior outcome (10.53 vs. 25.99; *p* = 0.0004) (Supplementary Figure S4B).

Taken together, the presence of somatic mutations in either *TP53*, *BCOR/BCORL1*, *SETBP1*, *RAS*, *EZH2*, or *DNMT3A* was associated with significantly inferior survival and hence labelled as “Adverse risk” mutations (7.3 vs. 26.0 months; *p* < 0.0001) (Figure 4B). Secondly, in the multivariate Cox proportional analysis, the Poor-risk group, derived from the machine learning model using clinical variables (HR 4.3, *p* < 0.0001), and Adverse risk mutations (HR 4.6, *p* < 0.0001) were independent risk factors for OS (Supplementary Figure S4C). Next, we determined whether the Adverse mutations could provide additional value to the risk group derived from the machine learning predictive model based on clinical variables. The median OS of Standard-risk patients harboring Adverse risk mutations was significantly shorter compared to patients without these Adverse risk mutations (16.3 vs. 43.5; *p* = 0.0015) (Supplementary Figure S4D). Similarly, Adverse risk mutations were significantly associated with shorter survival compared to non-Adverse risk mutations in the Poor-risk group (6.2 vs. 13.4 months; *p* = 0.013) (Supplementary Figure S4E). Finally, the integration of somatic mutation information into the prognostic model based on clinical variables identified three distinct groups of patients with significant survival differences between groups (6.2 vs. 16.3 vs. 43.5 months; *p* <0.0001) (Figure 4C and Supplementary Figure S4F).

As OS was significantly different in Adverse risk mutations, we compared the response to azacitidine according to individual somatic mutations. We did not find significant differences in the frequency of individual somatic mutations between responders and non-responders (Supplementary Results).

### 3.3. High Transfusion Burden and Healthcare Utilization in Poor-Risk Patients

**Figure 5:**
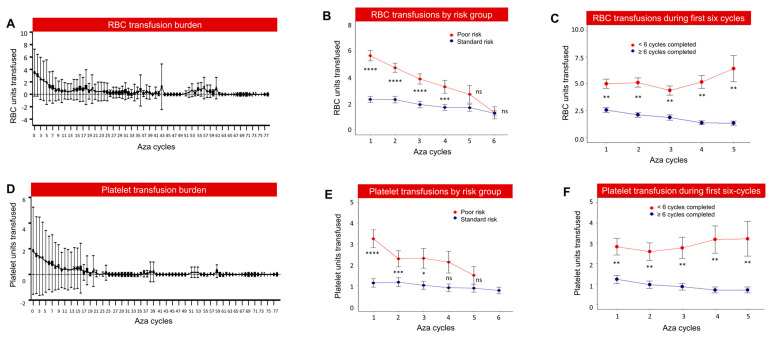
Transfusion burden during azacitidine therapy. (**A**) RBC transfusion burden is significantly high during initial 5 cycles. (**B**) RBC transfusion burden during initial five cycles was significantly higher in Poor-risk compared to Standard-risk patients. The data represent the mean ± the standard error measurement. (**C**) RBC transfusion burden continued to be higher in patients who are less likely to continue beyond six cycles. (**D**) Platelet transfusion burden remained high during initial 4–6 cycles of azacitidine therapy. (**E**) Platelet transfusion burden during first five cycles was significantly higher in Poor-risk compared to Standard-risk patients. (**F**) Platelet transfusion burden continued to be higher in patients who are less likely to continue beyond six cycles of azacitidine. The data represent the mean ± the standard error measurement (* represents *p* < 0.05, ** represent *p* < 0.01, *** represent *p* < 0.001, **** represent *p* < 0.0001, ns represents not significant).

We have previously shown that RBC-TD was associated with poor survival in MDS patients managed with supportive care [18]. Both RBC transfusion dependency and thrombocytopenia were identified as major contributors to OS in our ML model (Figure 1B). We assessed the impact of platelet and RBC transfusion on the outcome of azacitidine-treated patients and the subsequent impact of azacitidine therapy on transfusion requirements. RBC transfusion burden remained high during the initial six cycles of azacitidine therapy and decreased after that (Figure 5A). RBC transfusion burden was significantly higher in Poor-risk patients compared to Standard-risk ones (Figure 5B) and patients who stopped azacitidine during the first six cycles compared to patients who continued beyond six cycles (Figure 5C). Similarly, platelet transfusion demand was high during the first six cycles of azacitidine (Figure 5D). Platelet transfusion burden was significantly higher in Poor-risk patients compared to Standard-risk ones (Figure 5E) and in patients who stopped azacitidine therapy during the first six cycles compared to those who continued further (Figure 5F). Collectively, these data suggest that RBC and platelet transfusion demand remains high during the first six cycles of azacitidine especially in Poor-risk cases and patients not responding to azacitidine. Importantly, high RBC or platelet transfusion burden was associated with significantly poor OS compared to patients with lower transfusion burden (Supplementary Figure S5A–D).

Despite outpatient administration, hospitalization was required in 20% of cases during the first three cycles and ~10% of cases during subsequent cycles (Supplementary Figure S6A). Although hospitalization duration varied significantly during each cycle of azacitidine, the average hospitalization duration ranged between 5 and 10 days (Supplementary Figure S6B). Furthermore, more-frequent hospitalization was required in Poor-risk compared to Standard-risk patients (*p* = 0.002; Supplementary Figure S6C).

### 3.4. Poor-Risk Patients Were Less Likely to Complete Six Cycles of Azacitidine

**Figure 6:**
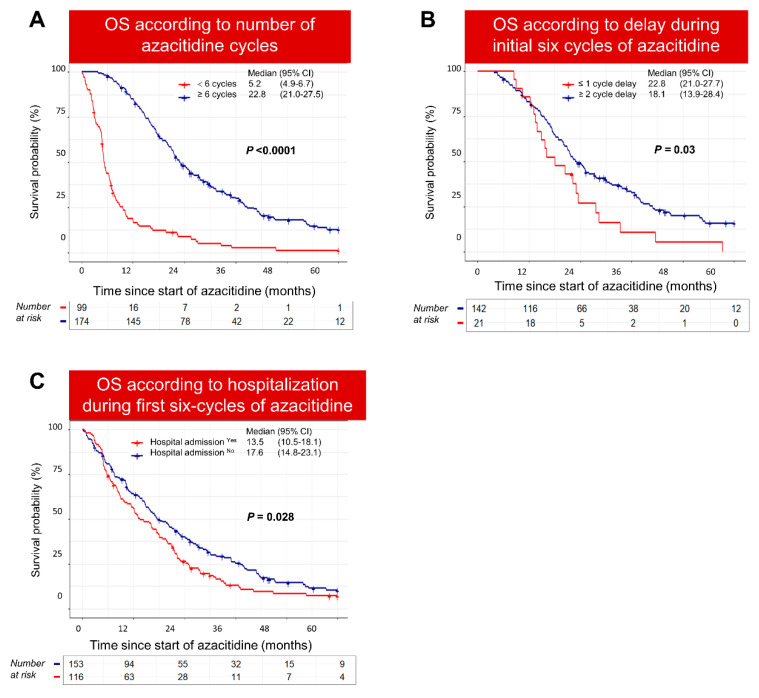
Overall survival was significantly poor in patients who did not complete six cycles of azacitidine. (**A**) Overall survival of patients who did not continue treatment beyond six cycles of azacitidine was significantly poor compared to patients who received more than six cycles. (**B**) Patients who continued treatment beyond six cycles and experienced a delay ≥ 2 azacitidine cycles, during the initial six cycles of azacitidine, had poorer survival compared to patients without such delays. (**C**) Patients who continued treatment beyond six cycles and required hospitalization during azacitidine treatment had significantly poor survival.

The median number of cycles completed was seven (range 1–78), and 75% (*n* = 206) and 60% (*n* = 174) of patients were able to complete four and six cycles of azacitidine, respectively. The median number of azacitidine cycles in the Poor-risk group (4; 95% CI 2.8, 8.0) was significantly lower than the Standard-risk group (9; 95% CI 5, 19) (*p* < 0.0001). Table S5 summarizes the baseline parameters in patients who completed versus those who did not complete six cycles of azacitidine. The most-common reasons for stopping azacitidine prior to six cycles were progressive disease or failure to respond (*n* = 45/96; 46.8%), death (*n* = 15; 15.8%), toxicity and infection (*n* = 17; 17.8%), patient preference (*n* = 6; 6%), and proceeding to definitive treatment with chemotherapy or allogeneic stem cell transplantation (*n* = 13; 13.6%). Importantly, the OS of patients who could not complete six cycles of azacitidine was significantly shorter compared to patients who continued treatment beyond six cycles (5.2 vs. 22.8 months; *p* < 0.0001) (Figure 6A).

We then restricted our survival analysis to patients who completed at least six cycles of azacitidine. Notably, patients who experienced delays between the cycles on two or more occasions during the first six cycles of azacitidine had significantly inferior survival compared to cases with no delays (18.1 vs. 22.8 months; *p* = 0.03) (Figure 6B). Similarly, patients who required hospitalization during the first six cycles had a worse outcome compared to patients who did not require hospitalization (13.5 vs. 17.6 months; *p* = 0.028) (Figure 6C).

### 3.5. Response to Azacitidine Did Not Completely Abrogate Inferior Survival of Poor-Risk Patients

**Figure 7:**
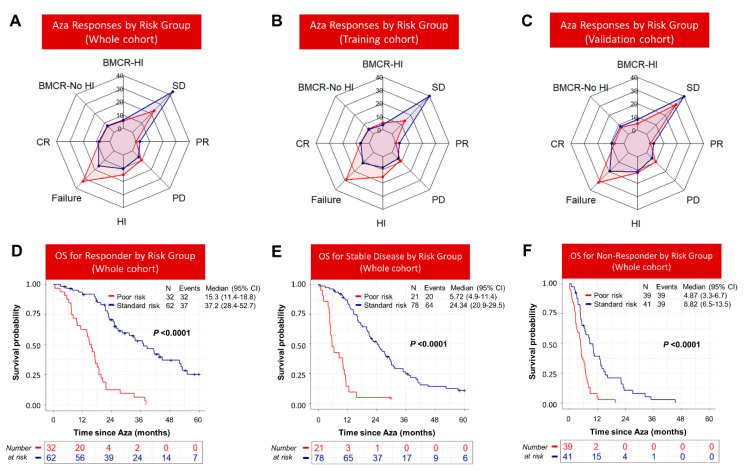
Responses following six cycles of azacytidine in Poor- and Standard-risk patients. Responses after six cycles of azacitidine in: (**A**) whole cohort; (**B**) training cohort; (**C**) validation cohort. The Poor-risk group is shown red color, while the Standard-risk group is shown in blue color. For each response category, the overall survival of Poor-risk patients was significantly poor compared to Standard-risk patients. (**D**) Responders; (**E**) stable disease; (**F**) non-responders.

In total, 94 (34.43%), 99 (36.3%), and 80 (29.3%) patients achieved any response, achieved stable disease, or were non-responders, respectively. Poor-risk patients were less likely to respond compared to Standard-risk ones (42.4% vs. 22.7%; *p* < 0.001) (Figure 7A–C), while Standard-risk patients were more likely to achieve stable disease (43.1% vs. 22.8%; *p* < 0.001) compared to Poor-risk patients (Figure 7A–C). Importantly, for each response category, the OS of Poor-risk patients was significantly inferior compared to Standard-risk patients. Poor-risk patients who responded (15.3 vs. 37.2 months; *p* < 0.0001), achieved stable disease (5.7 vs. 24.3 months; *p* < 0.0001), or did not respond (4.9 vs. 8.8 months; *p* < 0.0001) had significantly poor survival compared to Standard-risk patients within in each response category (Figure 7D–F). Similar findings were observed in both the training and validation cohorts (Supplementary Figure S7A–F). Collectively, these findings indicated that Poor-risk patients are less likely to respond to azacitidine, and their poor prognosis cannot be completely overcome by achieving a response after six cycles of azacitidine.

### 3.6. Poor Survival after Azacitidine Failure

At the time of last follow-up, 32% (87/273) of patients progressed to AML and 91% (238/273) of patients, including responders, eventually stopped azacitidine. Fourteen percent (39/273) of patients received further treatment including clinical trial, intensive chemotherapy, and allogenic stem cell transplant. Despite this, the median survival from cessation of azacitidine was 3.8 months (95% CI 3.16–4.61).

## 4. Discussion

We described a novel machine learning model, utilizing easily available baseline clinical variables and blood parameters, to predict survival outcome in High-risk MDS patients treated with azacitidine. The key findings of our study, based on real-life registry data, were: (i) azacitidine-treated patients experienced a low median OS of 16 months, shorter than reported in the pivotal AZA001 clinical trial [6], with a dismal 5-year OS of only 9%; (ii) Poor-risk patients, identified by our machine learning algorithm using clinical variables readily available in everyday practice, but not considered in decision-making, were unlikely to benefit from azacitidine; (iii) moreover, the integration of somatic mutation profiles stratified our model further to identify three distinct outcome subgroups with major and statistically significant differences in survival; (iv) finally, we illustrated the high healthcare utilization (including red cell and platelet transfusions and hospitalization requirement) of Poor-risk patients.

The identification of variables that would ascertain Poor-risk patients to prioritize for early allograft and/or participation in clinical trials is much needed [7,8,19,20]. To date, a limited number of risk models have focused specifically on outcomes related to HMA treatment. The widely used IPSS-R, already 10 years old [3], was derived from clinical parameters of patients receiving the best supportive care at the time.

Somatic mutations can predict the survival of MDS patients. We have previously shown that the *SF3B1* mutation, a spliceosome complex pathway gene, is associated with favorable outcome, while the *TP53* somatic mutation is associated with poor outcome in t-MN [21,22,23]. Furthermore, the median OS of patients with *TP53* wild-type (*TP53*^wt^) was significantly longer compared to *TP53*^mut^ (17.40 vs. 6.91; *p* < 0.0001). More recently, the Molecular International Prognostic Scoring System integrated clinical and mutation profiles of MDS patients, though only 19% of patients were treated with hypomethylating agents [24]. More recently, machine learning models have been applied to MDS; however, therapy was heterogenous with only 25% on HMA therapy [25]. Another machine learning model attempted to develop a biomarker gene combination predicting HMA resistance; however, it did not incorporate clinical characteristics, and the gene marker combination was present only in one-third of patients, limiting generalization [26]. Other models have utilized serial complete blood counts during the first 90 days of treatment to predict response to HMA. As this model relies on the change in serial blood counts over 90 days of treatment, it cannot assist in the upfront decision about whether to initiate HMA therapy. Secondly, this model does not predict survival outcome. Our model confirmed the predictive capacity of easily measurable clinical variables including LDH, blood counts, bone marrow blasts, RBC-TD status, age, and albumin. Interestingly, LDH was our top predictive variable and is not normally considered in the clinical management of MDS patients regarding treatment selection or prognosis. As a critical mediator of Warburg-type tumor metabolism, high levels of LDH likely reflect a high bioenergetic state including increased glycolytic activity and, presumably, increased cell turnover. Future studies should investigate LDH as a predictive marker in low blast count MDS.

We acknowledge the small sample size; however, our risk model was able to identify Poor-risk patients unlikely to benefit with azacitidine therapy. With increasing availability and the reduced cost of next-generation sequencing and expanding experience with ML techniques, such personalized medicine approaches will soon become standard of care. In summary, there is a substantial portion of Poor-risk group patients, identified by our machine learning model, that are unlikely to benefit from azacitidine, yet require a high degree of healthcare support. The validation of our findings on a large independent cohort will significantly increase the utility of this model. These findings are critical for optimizing the management of higher-risk MDS patients and the selection of HMA therapy or prompt consideration for molecularly defined clinical trials, such as novel therapies targeting BCL-2, IDH1/IDH2, or FLT-3 or allogeneic transplantation for fit patients.

The median OS of 16 months in our cohort is comparable to other real-world registries [8,9,10,11,12,13,14,25,27,28,29]. Several registry-based studies [1,7,10,11,28] consistently reported a shorter survival benefit with azacitidine compared to AZA-001 [6] (11–16 months vs. 24 months). More recently, pooled data from clinical trials with azacitidine monotherapy furthered this real-life observation with a new benchmark of 18.6 months [29]. The only study to achieve a median OS of 27 months included all MDS patients, and impressively, 70% of patients were lower-risk MDS [11]. Our study is one of the few assessing long-term outcomes, with a five-year OS of higher-risk MDS of only 9%. Similar dismal five-year OS probability rates of 4% (95% CI 2–6%) were reported from the Surveillance, Epidemiology, and End Results (SEER)-Medicare-linked database [12]. The SEER report, however, did not have patient-level details available, did not provide information on MDS risk as per IPSS-R, and restricted analysis to patients older than 65 years [28]. Reasons cited for the survival disparity between the pivotal Aza-001 trial and real-world analysis include the younger age and relative fitness of the included patients, a variation in the azacitidine schedule, the persistence of therapy after response, and the exclusion of therapy-related myeloid neoplasm (t-MN) patients [19]. In the AZA-001 trial, 24% of patients (87/358) were ≥75 years of age, while in our study, 40% (111/274) of patients were ≥75 years of age. Similarly, 51% of patients enrolled in the AVIDA registry were ≥ 75 years and 30% were ≥80 years, and the median age in the Canadian registry was 74 years (19–99) compared to 69 (42–83) years in the pivotal trial. The Canadian registry population was more likely to harbor Poor-risk cytogenetics (38%), almost identical to our 39%, versus 28% in AZA-001 [6].

## 5. Conclusions

Our study demonstrated the poor long-term survival of higher-risk MDS patients treated with azacitidine and adds to the existing evidence that azacitidine monotherapy is inadequate for many MDS patients. We, therefore, recommend the consideration of up-front enrolment into clinical trials and/or consideration for allogeneic SCT, rather than defaulting to the routine use of standard of care.

## Data Availability

The data that support the findings of this study are available from the corresponding author upon reasonable request.

## Supplementary Materials

The following supporting information can be downloaded at: https://www.mdpi.com/article/10.3390/cancers15164019/s1, Table S1: Clinical features and demographic of the MDS treated with azacitidine. Table S2: IPSS and IPSS-R of de novo MDS and oligoblastic AML at treatment. Table S3: Patient characteristics for training and validation cohorts. Table S4: Clinical and cytogenetic variables included in the machine learning model. Table S5: Baseline clinical variables in patients who completed and could not complete six-cycles of azacitidine. Figure S1: Schematic of machine learning approach and its performance. Figure S2: Overall survival according to chromosome 19 abnormality and neutrophils counts in (A,B) training and (C,D) validation cohorts. Figure S3. Performance of the prognostic model, derived by machine learning, and IPSS-R for predicting overall survival of azacitidine treated patients. Figure S4. Integration of somatic mutation profile refine the machine learning (ML) model. Figure S5: Overall survival of azacitidine treated patients according to transfusion burden. Figure S6. Hospitalization during azacitidine therapy. Figure S7: In each response category, overall survival of Poor risk patients was significantly inferior compared to Standard risk patients in (A–C) Training cohort and (D–F) Validation cohort. (Reference [30] was cited in the Supplementary Materials).
