# Supplementary material for: A Personalized Risk Model for Azacitidine Outcome in Myelodysplastic Syndrome and Other Myeloid Neoplasms Identified by Machine Learning Model Utilizing Real-World Data
Source: Cancers (Basel). 2023 Aug 8;15(16):4019. doi: 10.3390/cancers15164019 (PMC10452100; doi:10.3390/cancers15164019)
Supplement: Supplementary file 1 [file cancers-15-04019-s001.zip › cancers-2446831-supplementary.pdf]

A personalized risk model for azacitidine outcome in myelodysplastic syndrome and other myeloid neoplasm identified by machine-learning model utilizing real-world data

This file contains:
Supplementary Methods

Tables S1 to S5

Figures S1 to S7

Supplementary Methods

Random survival forest machine learning algorithm

Random survival forests (RSF)[1] is an extension of random forest algorithm but apply to the setting of right-censored survival data. RSF has same general principles as random forest: (a) Survival trees are grown using bootstrapped data i.e. 0.632 sampling without replacement; (b) Random feature selection is used when splitting the tree nodes; (c) trees are generally grown deeply, and (d) the survival forest ensemble is calculated by averaging terminal node statistics (TNS). The TNS is the Kaplan-Meier estimator and the Nelson-Aalen cumulative hazard function. RSF returns an ensemble predicted mortality value for each sample which is calculated using the TNS for the sample. The mortality value (risk score)[1] represents estimated risk for each individual calibrated to the scale of the number of events. For example, if i has a mortality value of 100, then if all individuals had the same covariate as i, which is X=xi, we would expect an average of 100 events. RSF return out-of-sample predicted values which are calculated using the hold out data for each tree. The out-of-sample values are used for inference on the training data. This is because they are cross-validated and will not over-fit the data. Prediction error for survival models is measured by 1−C, where C is Harrell's concordance index[2] to assess prediction performance in survival settings. Prediction error is between 0 and 1, and measures how well the predictor correctly ranks two random individuals in terms of survival. RSF returns out-of-sample prediction error. RSF provides a fully nonparametric measure of variable importance (VIMP). VIMP is a technique for estimating the importance of a variable by comparing performance of the estimated model with and without the variable in it. For further details please refer to Ishwaran et al. (2021)[3].

Supplementary results

Neutrophil counts prior to starting azacitidine was not significantly different between MDS, AML and CMML: Patients were classified into MDS Low risk (n=4), Intermediate risk (n=39), High risk (n=100) as per IPSS-R classification. The study also included AML (n=61), t-MN (n=45), and CMML (n=24). The mean±SD (standard deviation) of neutrophil counts of Low risk (1.10±0.61), Intermediate risk (4.37±10.99), High risk (3.92±8.66), AML (4.07±6.88), t-MN (2.39±5.01), and CMML (8.65±14.22) was not significantly different between the groups (p=0.202). This suggests that neutrophil counts may not be a distinguishing factor for risk stratification in our cohort.

Frequency of abnormal chromosome 19 was not significantly different between MDS, AML and CMML:

Moreover, frequency of abnormal chromosome in Low risk (25%), Intermediate risk (0%), High risk (9.2%), AML (5.1%), t-MN (11.4%), and CMML (4.2%) was not significantly different between the groups (p=0.24).

Azacitidine response rate according to somatic mutation type: Role of somatic mutations in predicting response to azacitidine is actively debated. We observed a higher frequency of *TP53* (42% vs. 24%; *P* =0.09), *SETBP1* (9.7% vs. 4.8%; *P*=0.41), and *DNMT3A* (26% vs. 14%; *P =0.21*) mutations in the non-responder compared to the responder group, although these differences did not reach statistical significance. While frequency of *ASXL1* (31% vs. 16%; *P* =0.14), *IDH1/2* (12% vs. 7%; *P* =0.43), *SRSF2* (31% vs. 19%; *P*=0.26), and *TET2* (36% vs. 29%; *P* =0.54) mutations, although none of the differences were statistically significant. This could be due to limited sample size and further studies including larger cohort of cases would be beneficial.

SUPPLEMENTARY TABLES

Table S1: Clinical features and demographic of the MDS treated with azacitidine

Variables	(*n* = 273)
**Clinical features at diagnosis**	
Age, median (IQR)	73 (67 - 78)
Male/female	191/82
**Charlson Co-morbidity Index**	***n* = 273**
1	4 (2)
2	65 (24)
3	119 (43)
>4	85 (31)
**Rural/Urban**	**71/202**
**WHO subcategories at diagnosis, *n* (%)**	
AML	50 (18)
MDS EB1	23 (8)
MDS EB2	100 (37)
MDS SLD/MDS MLD	17 (6)
t-MN	59 (22)
CMML	24 (9)
**IPSS-R, *n=185* (%)**	
Very low	2 (1)
Low	3 (2)
Intermediate	31 (17)
High	68 (37)
Very High	81 (44%)
**Karyotype, *n* (%)**	
Very Good	6 (2)
Good	101 (37)
Intermediate	52 (19)
Poor	30 (12)
Very poor	74 (27)
Missing	10 (3)
**Bone marrow blast at start azacitidine *n* (%)**	
<5%	42 (15)
5-10%	45(14)
11-20%	123 (46)
>20%	57 (23)
Missing	6(2)
**Platelet count prior to starting azacitidine**	
< 50 $x10^9$/L	128 (47)

> 50 x10^9/L	145 (53)
**RBC units transfused ≥ 4 units 8 weeks pre-azacitidine**	
Yes	103 (38)
No	170 (62)
**Number of cytopenia**	
0	10 (3)
1	82 (30)
2	112 (41)
3	67 (25)
Missing	2 (0.007)

Table S2: IPSS and IPSS-R of *de novo* MDS and oligoblastic AML at treatment

		R-IPSS				
		**Very low (*n* = 2)**	**Low (*n* = 3)**	**Intermediate (*n* = 31)**	**High (*n* = 68)**	**Very High (*n* = 81)**
**IPSS**	Low (*n* = 2)	2 (100%)	0 (0%)	0 (0%)	0 (0%)	0 (0%)
	Int-1 (*n* = 26)	0 (0%)	3 (11%)	15 (58%)	8 (31%)	0 (0%)
	Int-2 (*n* = 91)	0 (0%)	0 (0%)	14 (16%)	43 (47%)	34 (37%)
	High (*n* = 66)	0 (0%)	0 (0%)	2 (3%)	17 (26%)	47 (71%)
**Total**	185	2 (1%)	3(1%)	31 (17%)	68 (37%)	81 (44%)

IPSS and IPSS-R was not assessed in t-MN (n=59) and proliferative CMML and MDS/MPN-U (n=23) as these patients were excluded in the original IPSS and IPSS-R prognostic scoring system

Table S3: Patient characteristics for training and validation cohorts

Characteristics	Overall (n = 273)	Training (n= 139)	Validation (n= 134)	*P*
**Age, median (IQR)**	73 (67, 78)	72 (67, 77)	73 (67, 79)	0.231
**SEX *n* (%)**				
Male	191 (70)	96 (69.1)	95 (70.9)	0.792
Female	82(30)	43 (30.9)	39 (29.1)	
Interval from diagnosis to HMA start, median (IQR)	2.20 (0.92, 11.51)	1.81 (0.90, 10.45)	2.29 (1.09, 11.58)	0.629
**Pre-Treatment Clinical Variables, median (IQR)**				
Hemoglobin	92.00 (83, 104)	91 (82.5,106.2)	92 (84.1,101.0)	0.807
Neutrophil Count	1.39 (0.67,3.10)	1.47 (0.78, 3.63)	1.33 (0.54, 2.78)	0.182
Platelet count	58 (31,101)	46 (30.5,103.2)	64.5(32,95.3)	0.579
Bone Marrow Blast Count	12 (8,18)	12 (8,19)	12 (8,18)	0.41
Creatinine	86 (72, 106)	87 (71.5, 106)	86 (72, 106.2)	0.84
Albumin	36 (33, 39)	37 (34, 39)	36 (33, 39)	0.422
Bilirubin	11 (8,16)	11 (8, 17)	11 (8, 16)	0.879
LDH	244 (200, 329)	253 (203.5, 334.2)	239 (196, 307)	0.266
ALT	20 (14, 29)	18 (13, 27)	21 (15, 30)	0.075
GGT	32.5 (22, 59)	33 (22, 59)	32 (22, 59)	0.953
RBC units transfused from diagnosis to HMA start, median (IQR)	4 (0, 10.2)	4 (0, 11.7)	4 (0, 10)	0.878
Number of platelet units transfused, median (IQR)	0 (0, 2)	0 (0, 2)	0 (0, 2)	0.21
**RBC Transfusion Dependency *n* (%)**				
Dependent	113 (43.8)	61 (46.2)	52 (41.3)	0.453
Independent	145 (56.2)	71 (53.8)	74 (58.7)	
**WHO subtype *n* (%)**				
MDS-SLD	4 (2)	2 (1.4)	2 (1.5)	0.863
MDS-MLD	21 (8)	8 (5.8)	13 (9.7)	
MDS-EB1	36 (13)	20 (14.5)	16 (11.9)	
MDS-EB2	124 (46)	63 (45.7)	61 (45.5)	

AML (blasts 20-30%)<br>MDS/MPN overlap	62 (23)<br>25 (9)	33 (23.9)<br>12 (8.7)	29 (21.6)<br>13 (9.7)	
**De novo MDS, *n* (%)**<br>**t-MN n (%)**	215 (78.8)<br>58 (21.2)	110 (79.1)<br>29 (20.9)	105 (78.4)<br>29 (21.6)	0.884
**IPSS-R prior to starting azacitidine n (%)**				
Low	9 (3.3)	5 (3.6)	4 (3.0)	1
Intermediate	79 (29.0)	40 (29.0)	39 (29.1)	
High	89 (32.7)	45 (32.6)	44 (32.8)	
Very High	95 (34.9)	48 (34.8)	47 (35.1)	
**Place of residence n (%)**				
Rural	70 (25.6)	38 (27.3)	32 (23.9)	0.58
City	203 (74.4)	101 (72.7)	102 (76.1)	
Chromosome 3 abnormality *n* (%)	27 (10.2)	15 (11.3)	12 (9.0)	0.685
Chromosome 5 abnormality *n* (%)	63 (23.7)	29 (21.8)	34 (25.6)	0.564
Chromosome 7 abnormality *n* (%)	67 (25.2)	41 (30.8)	26 (19.5)	0.048
Trisomy 8 *n* (%)	43 (16.2)	21 (15.8)	22 (16.5)	1
Chromosome 9 abnormality *n* (%)	18 (6.8)	7 (5.3)	11 (8.3)	0.465
Chromosome 11 abnormality *n* (%)	31 (11.7)	16 (12.0)	15 (11.3)	1
Chromosome 12 abnormality *n* (%)	36 (13.6)	17 (12.9)	19 (14.3)	0.858
Chromosome 13 abnormality *n* (%)	24 (9.1)	12 (9.1)	12 (9.0)	0.831
Chromosome 16 abnormality *n* (%)	20 (7.5)	11 (8.3)	9 (6.8)	0.817
Chromosome 17 abnormality *n* (%)	35 (13.3)	16 (12.2)	19 (14.4)	0.717
Chromosome 18 abnormality *n* (%)	23 (8.6)	10 (7.5)	13 (9.8)	0.663
Chromosome 19 abnormality *n* (%)	19 (7.2)	12 (9.1)	7 (5.3)	0.245
Chromosome 20 abnormality *n* (%)	31 (11.7)	17 (12.9)	14 (10.5)	0.572
Chromosome 21 abnormality *n* (%)	19 (7.2)	11 (8.3)	8 (6.0)	0.486
Deletion Y *n* (%)	11 (4.2)	5 (3.8)	6 (4.5)	1
Complex karyotype n (%)	66 (24.8)	32 (24.1)	34 (25.6)	0.887
Monosomal karyotype n (%)	54 (20.3)	28 (21.1)	26 (19.5)	0.879
Complex and monosomal karyotype *n* (%)	47 (17.7)	24 (18.0)	23 (17.3)	1
Marker chromosome n (%)	36 (13.6)	16 (12.1)	20 (15.0)	0.591
Ring chromosome *n* (%)	9 (3.4)	4 (3.0)	5 (3.8)	1

Table S4: Clinical and cytogenetic variables included in the machine learning model

Clinical variables	Cytogenetics variables
**SEX**	Chromosome 3 abnormality
**Charlson co-morbidity index**	Chromosome 5 abnormality
**RBC transfusion dependency status**	Chromosome 7 abnormality
**WHO subtype**	Trisomy 8
**therapy-related MDS**	Chromosome 9 abnormality
**Place of residence**	Chromosome 11 abnormality
**Age**	Chromosome 12 abnormality
**Hemoglobin**	Chromosome 13 abnormality
**RBC units transfused from diagnosis to HMA start**	Chromosome 16 abnormality
**Platelet count**	Chromosome 17 abnormality
**Number of platelet units transfused**	Chromosome 18 abnormality
**Neutrophil count**	Chromosome 19 abnormality
**Bone marrow blast percentage**	Chromosome 20 abnormality
**Interval between diagnosis and starting azacitidine**	Chromosome 21 abnormality
**Creatinine**	Chromosome Y deletion
**Albumin**	Complex karyotype
**Bilirubin**	Monosomal karyotype
**LDH**	Complex plus monosomal karyotype
**ALT**	Marker chromosome
**GGT**	Ring chromosome

Table S5: Baseline clinical variables in patients who completed and could not complete six-cycles of azacitidine

Variables	≥ 6 cycles completed (*n* = 174)	< 6 cycles completed (*n* = 99)	*P*-value
Age at diagnosis	71.5 (67 - 78)	74 (53 - 87)	0.42
Sex M/F	120/44	71/38	0.233
**R-IPSS prior to starting azacitidine**			
Very Low	2 (1%)	0 (0%)	0.001
Low	6 (3%)	1 (1%)	
Intermediate	55 (32%)	24 (24%)	
High	63 (36%)	26 (26%)	
Very High	47 (27%)	48 (49%)	
**Bone marrow blasts prior to starting azacitidine**			
0-5%	28 (16%)	14 (14%)	0.98
5-10%	25 (14%)	14 (14%)	
11-20%	81 (47%)	47 (48%)	
>20%	40 (23%)	24 (28%)	
**MDS risk group according to WHO classification**			
MDS-EB1	13 (8%)	20 (20%)	0.49
MDS-EB2	68 (39%)	27 (27%)	
AML	26 (15%)	15 (15%)	
t-MN	32 (18%)	27 (27%)	
CMML	19 (11%)	5 (5%)	
Other MDS	16 (9%)	5 (5%)	
**Blood counts and RBC transfused prior to starting azacitidine**			
Platelet count < $50x10^9$/L	67 (38%)	61 (61%)	**0.002**
RBC units transfused > 4	58 (33%)	55 (55%)	**<0.001**

Supplementary Figures

Figure S1

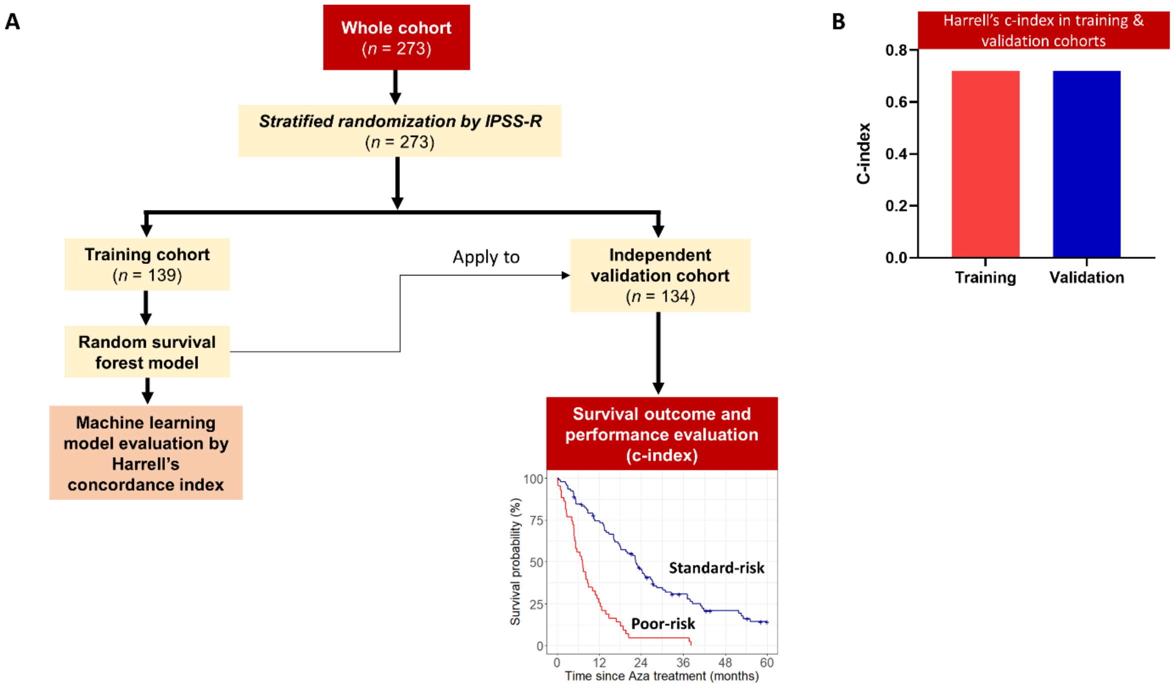

Figure S1: Schematic of machine learning approach and its performance. (A) The schematic of Machine learning approach. (B) The performance of the model measured by Harell's concordance index in training and validation cohort.

Figure S2

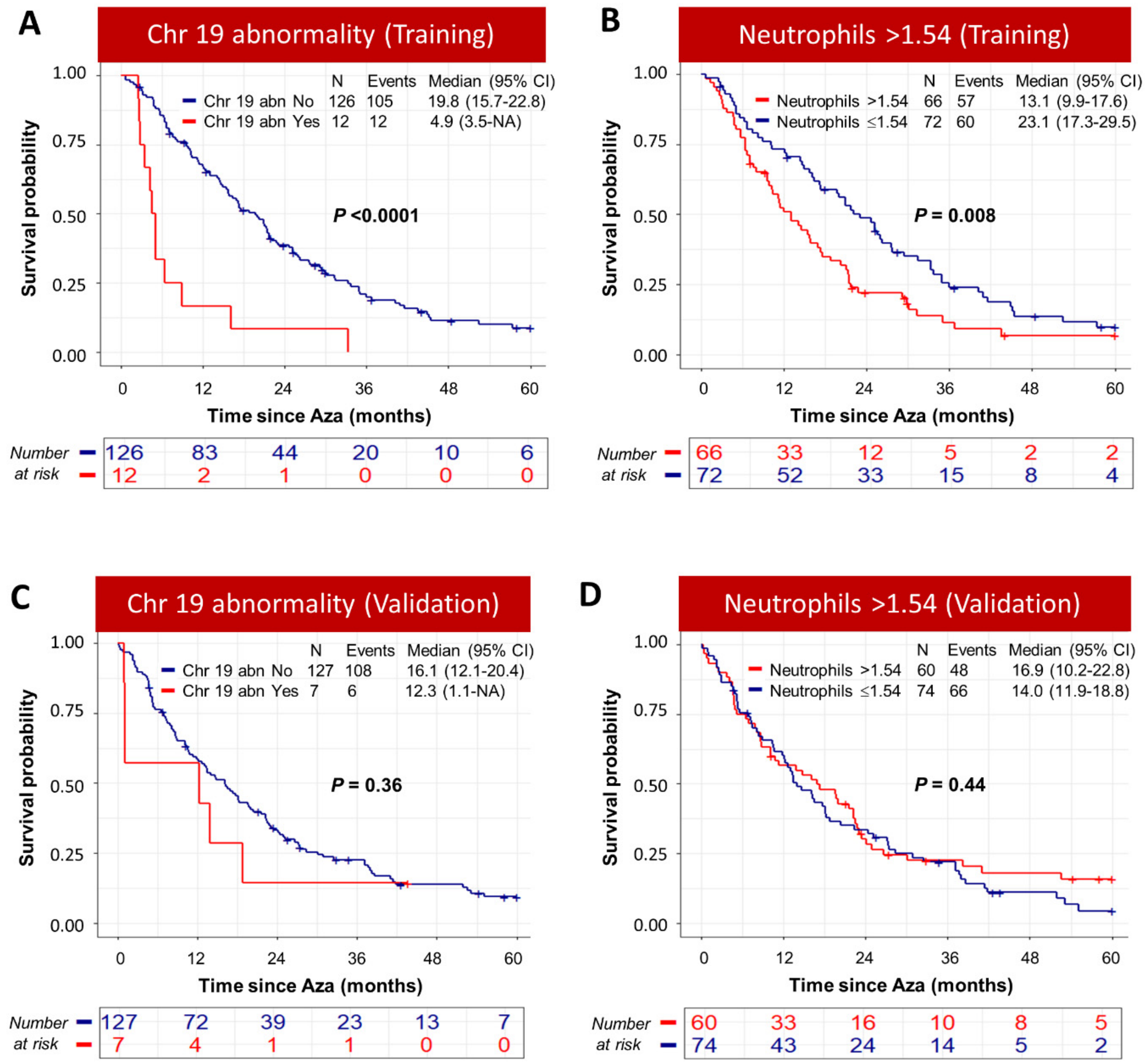

Figure S2: Overall survival according to chromosome 19 abnormality and neutrophils counts in (A-B) training and (C-D) validation cohorts.

Figure S3

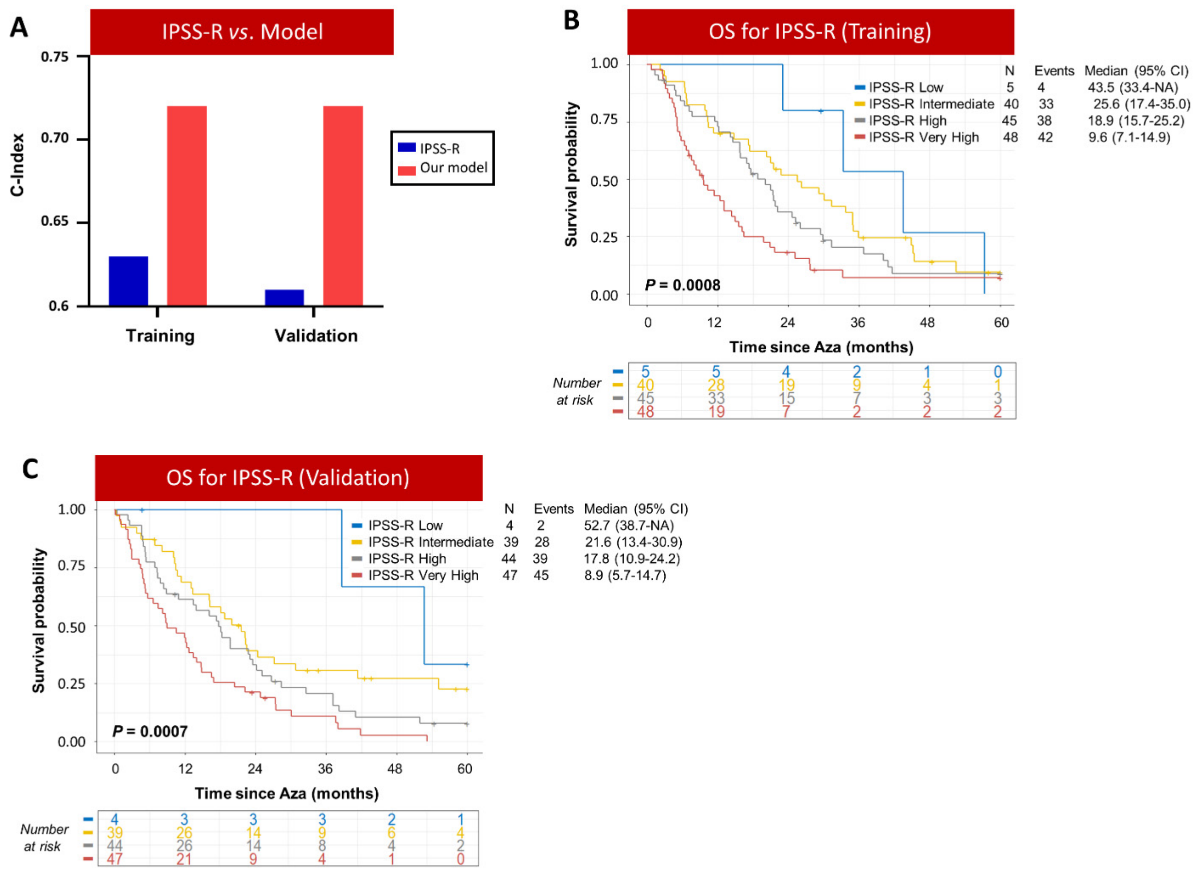

Figure S3. Performance of the prognostic model, derived by machine learning, and IPSS-R for predicting overall survival of azacitidine treated patients. (A) The concordance index (c-Index), a commonly used metric to evaluate how good prediction model is, for the machine learning model was 0.72 in the training and validation cohort, while 0.62 and 0.61 for the IPSS-R in the training and validation cohorts, respectively. Overall survival according to IPSS-R risk group in the (B) training and (C) validation cohorts.

Figure S4

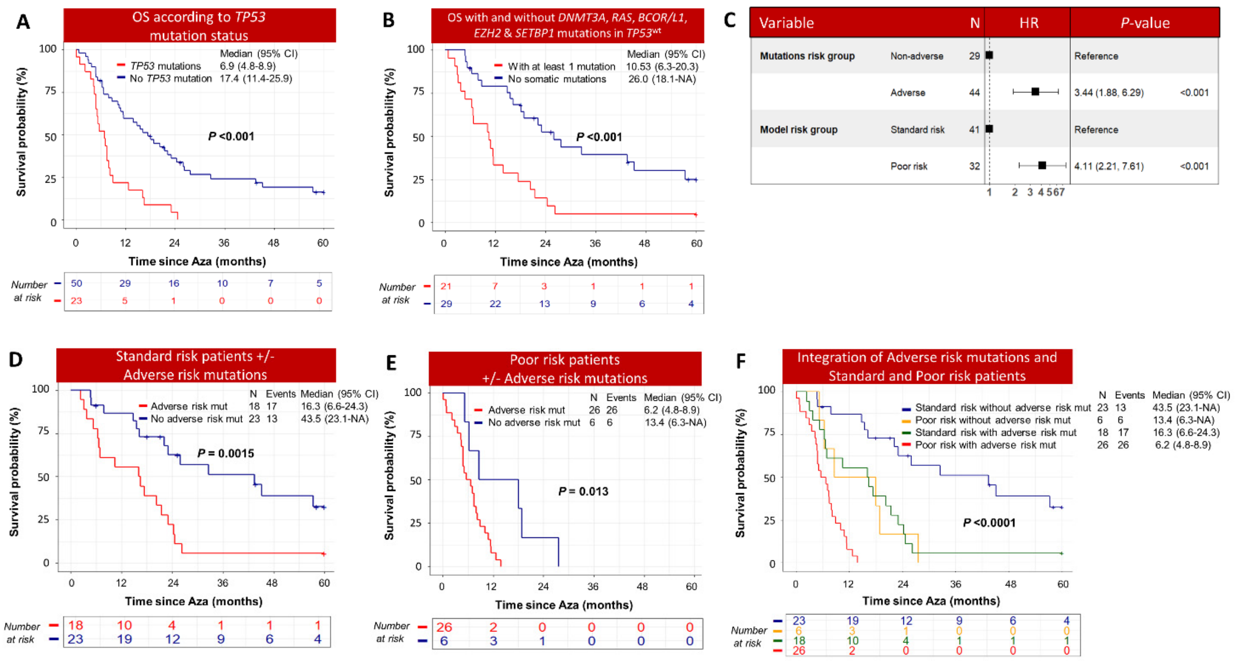

Figure S4. Integration of somatic mutation profile refine the machine learning (ML) model. (A) Overall survival of *TP53* mutated patients was significantly poor compared to patients without *TP53 mutation;* (B) In patients without *TP53* mutation (*TP53*^{wt}), *DNMT3A*, *RAS*, *BCOR*, *BCORL1*, *EZH2* or *SETBP1* mutation was associated with poor survival; (C) Forest plot shows the multivariate Cox proportional hazard regression analysis on mutational risk groups and the ML model risk groups. (D) Adverse risk mutations (*TP53*, *DNMT3A*, *RAS*, *BCOR*, *BCORL1*, *EZH2* or *SETBP1)* stratified Standard risk patients further with distinct survival difference; (E) OS of Poor risk patients with and without Adverse risk mutations; (F) Integration of Adverse risk mutations and risk group identified by clinical variables using ML algorithm.

Figure S5

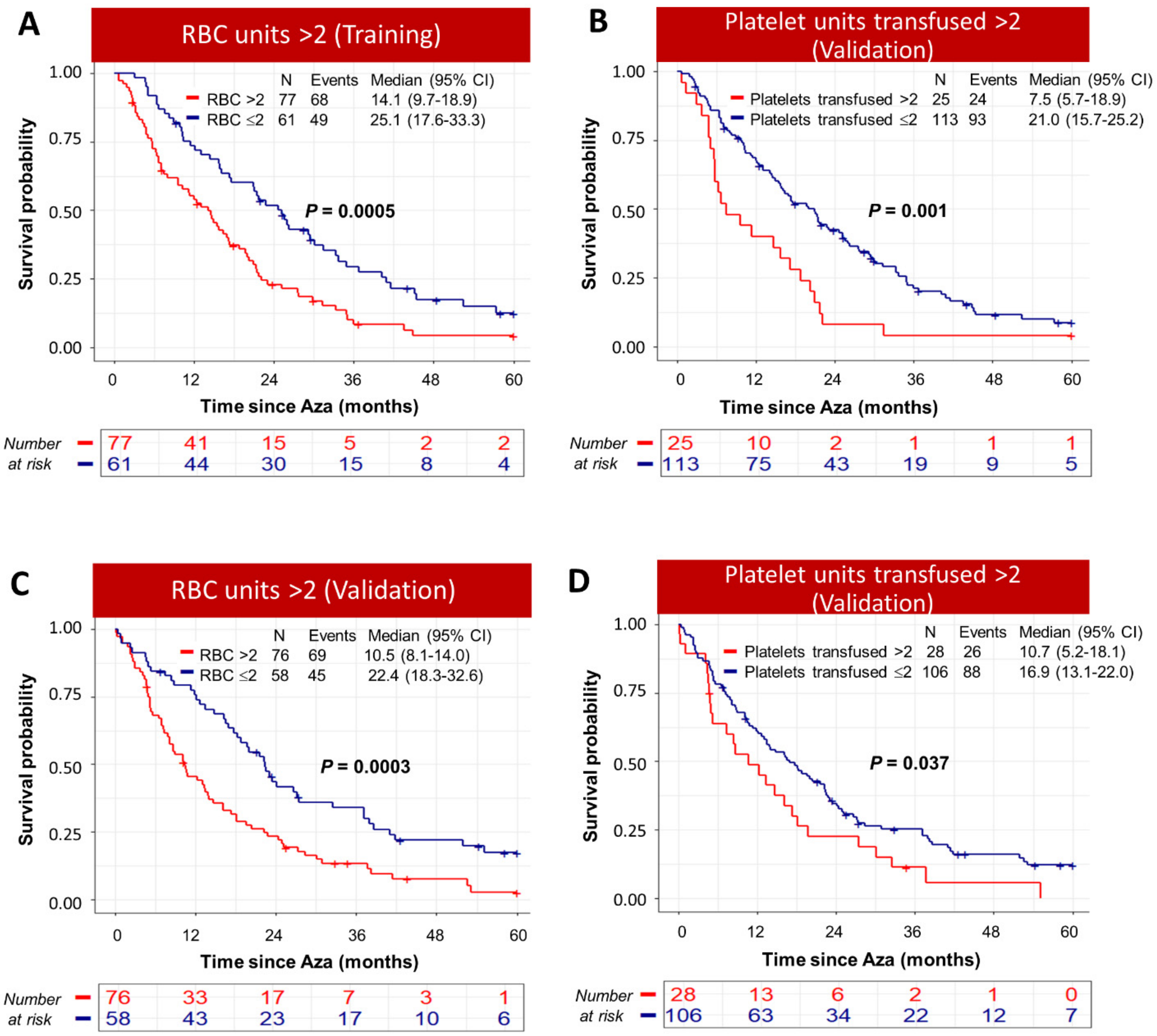

Figure S5: Overall survival of azacitidine treated patients according to transfusion burden. RBC-transfusion burden is associated with poor outcome in the (A) training and (B) validation cohort. Similarly, platelet transfusion is associated with poor survival in the (C) training and (D) validation cohort.

Figure S6

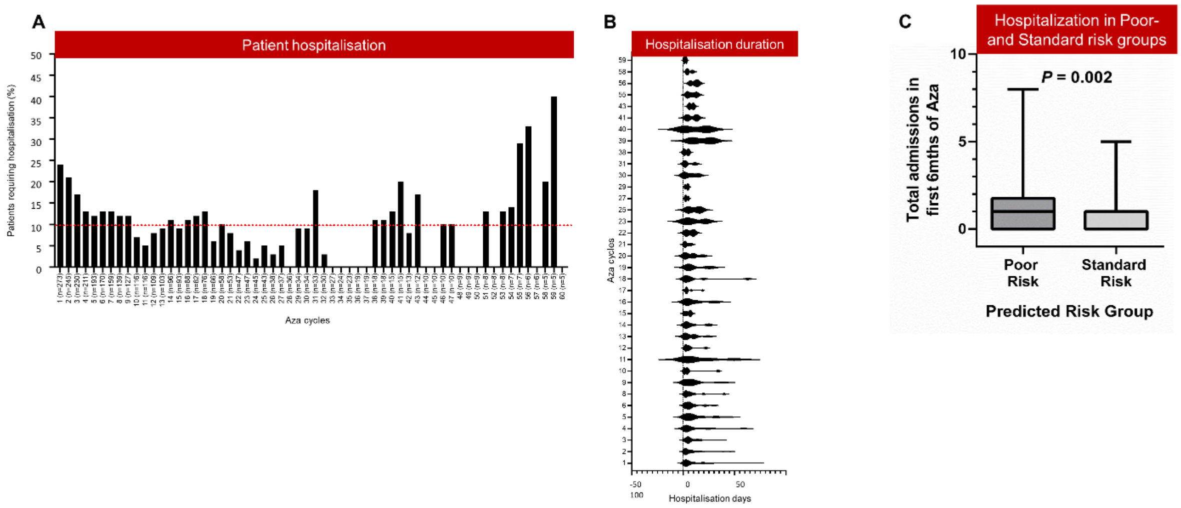

Figure S6. Hospitalization during azacitidine therapy. (A) Bar diagram showing percentage of patients requiring hospitalization during each cycle of azacitidine; (B) duration of hospitalization during each cycle of azacitidine; (C) hospitalization burden was higher in Poor risk compared to Standard risk group.

Figure S7

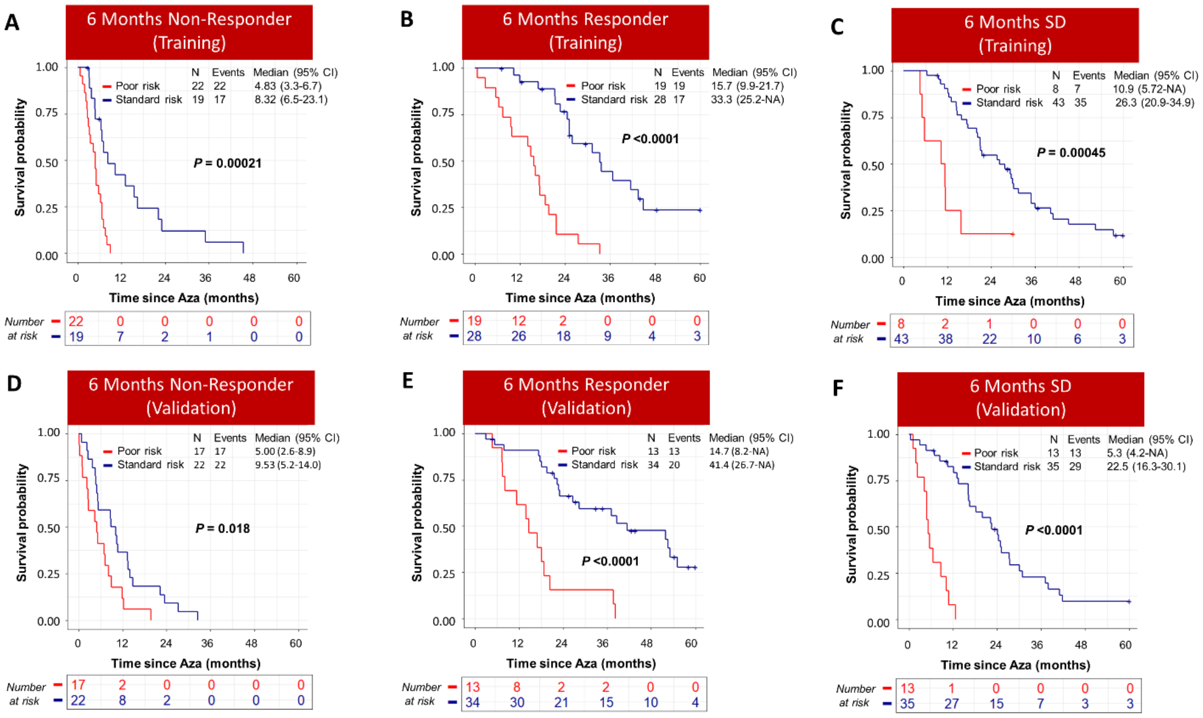

Figure S7: In each response category, overall survival of Poor risk patients was significantly inferior compared to Standard risk patients in (A-C) Training cohort and (D-F) Validation cohort.

References

1. Ishwaran H, Kogalur UB, Blackstone EH, Lauer MS. Random survival forests. The Annals of Applied Statistics. 2008;2(3):841-860, 820.
2. Harrell FE, Jr, Califf RM, Pryor DB, Lee KL, Rosati RA. Evaluating the Yield of Medical Tests. JAMA. 1982;247(18):2543-2546.
3. Ishwaran H, Lauer MS, Blackstone EH, Lu M, Kogalur UB. "randomForestSRC: random survival forests vignette." http://randomforestsrc.org/articles/survival.html. 2021;
